# Emerging of antimicrobial resistance in staphylococci isolated from clinical and food samples in Algeria

**DOI:** 10.1186/s13104-018-3762-2

**Published:** 2018-09-12

**Authors:** Rachid Achek, Helmut Hotzel, Zafer Cantekin, Ibrahim Nabi, Taha Mossadak Hamdi, Heinrich Neubauer, Hosny El-Adawy

**Affiliations:** 1 Faculty of Sciences, Yahia Farès University, Urban Pole, Médéa, Algeria; 2 High National Veterinary School, Issad Abbes Avenue, Oued Smar, Algiers, Algeria; 3 Department of Microbiology, Faculty of Veterinary Medicine, Mustafa Kemal University, Tayfur Sokmen Campus, 31000 Hatay, Turkey; 4 Institute of Bacterial Infections and Zoonoses, Friedrich-Loeffler-Institut, Naumburger Str. 96a, 07743 Jena, Germany; 5 Department of Poultry Diseases, Faculty of Veterinary Medicine, Kafrelsheikh University, Kafr El-Sheikh, 35516 Egypt

**Keywords:** *Staphylococcus aureus*, MRSA, Coagulase-negative staphylococci, Antimicrobial resistance, Algeria

## Abstract

**Objective:**

The antimicrobial resistance of staphylococci rose worldwide. In total, 96 *Staphylococcus* isolates from food and clinical samples were collected from two provinces in Algeria. The antimicrobial susceptibility testing was performed and resistance-associated genes were detected.

**Results:**

Fifty-one strains were isolated from food samples and differentiated into 33 *Staphylococcus aureus* and 18 coagulase-negative staphylococci. Forty-five staphylococci were collected from hospital and community-acquired infection cases. All *S. aureus* isolated from food were resistant to penicillin and 45.5% were resistant to tetracycline. The resistance rates of 45 clinical *Staphylococcus* isolates were 86.7%, 48.9%, 37.8% and 20.0% to penicillin, tetracycline, erythromycin and kanamycin, respectively. Nine isolates were confirmed as MRSA from food and clinical isolates. One *S. aureus* originated from food was confirmed as vancomycin-resistant. Multidrug-resistance was observed among 25.5% and 53.3% of food and clinical staphylococci, respectively. The *tet*M/K, *bla*Z, *aac*A-*aph*D, *erm*C and *mec*A genes were detected in food and clinical isolates. *erm*A gene was not found. This study provided insight into the status of antimicrobial resistance of staphylococci isolated from food and clinical samples in Algeria. Further investigations and surveillance programmes are mandatory.

## Introduction

Staphylococci are the most isolated bacteria in nosocomial infections and foodborne illnesses globally and involved in severe systemic affections [1–6].

The mechanism of antimicrobials resistance in staphylococci was due to the acquisition of mobile genetic elements like plasmids and/or transposons [7–10]. Penicillin-resistance was due to the production of beta-lactamases which is encoded by the *bla*Z gene located chromosomally or on plasmids [11, 12]. Methicillin-resistant *Staphylococcus aureus* (MRSA) where resistance is encoded by *mec*A gene is an important cause of human nosocomial infections worldwide [13, 14]. In *S. aureus* and CoNS tetracycline resistance is mediated by ribosomal encoded *tet*M gene and/or by *tet*K encoded efflux protein [15–17]. Ribosomal target modification, mediated by the presence of *erm*A*, erm*B and *erm*C in *S. aureus* and CoNS is associated with resistance to macrolides, lincosamides and type B streptogramins [17, 18]. For aminoglycoside resistance *aac*A-*aph*D genes are conferring cross-resistance to aminoglycosides such as gentamicin, tobramycin, kanamycin and amikacin [19–21].

Food is considered as an important vehicle for the spread of antibiotic-resistant bacteria [6, 22]. In meat-producing animals such as cattle, poultry and swine, antibiotics are mainly used for the treatment and prevention of several bacterial diseases which may lead to arise of antimicrobial resistance in various bacteria like *Campylobacter*, *Escherichia coli*, *Salmonella* and *Staphylococcus* [23].

In Algeria, antimicrobial susceptibility of *S. aureus* was commonly investigated. [24–26]. Less attention was given to *S. aureus* and CoNS in community-acquired infections or those isolated from food samples [27].

The aim of this study was to determine phenotypic antimicrobial resistance and associated genes of staphylococci isolates from clinical and food samples in Algeria.

## Main text

### Methods

#### Sample collection and processing

One hundred and twelve food samples, including raw milk (n = 30), minced beef meat (n = 25), chicken meat (n = 18), creamy cake (n = 14), pizza (n = 10), beef meat (n = 10) and sausages (n = 5) were collected from retail markets in cities of Médéa and Ain Defla provinces, Algeria.

Forty-five *S. aureus* isolates from clinical samples like pus, sperm, urine, vaginal discharge, wounds, catheter tips and secretions were kindly provided from Mohamed Boudiaf hospital, Médéa.

#### Bacterial isolation and identification

Microbiological analysis of food samples was done according to NF EN ISO 6888-1/A1 standard procedure according to the IOS [28] and for clinical samples was done by the method described previously [29]. The identification of coagulase-positive *S. aureus* (CoPS) was made by biochemical tests (Rabbit plasma, Oxoid, Dardilly, France). Identity of *S. aureus* was confirmed by agglutination test (Bio-Rad, Marnes-la-Coquette, France).

#### Susceptibility test of antimicrobial agents

The antimicrobial susceptibility testing to 12 antimicrobial agents was performed using the disc diffusion test according to CLSI recommendations [30] and the guidelines established by the Antibiogram Committee of the French Microbiology Society [31]. The antibiotic discs (Bio-Rad; Oxoid) were used according to instructions of the manufacturer’s (Table 1). *Staphylococcus aureus* ATCC 25923 was used as quality control.

**Table 1:** Antimicrobial susceptibility of staphylococci isolated from food and clinical samples

Antibiotic agent	Food isolates (n = 51)						Clinical isolates (n = 45)								
							Hospital acquired isolates (n = 18)			Community acquired isolates (n = 27)					
	*S. aureus* (n = 33)			CoNS (n = 18)			*S. aureus* (n = 18)			*S. aureus* (n = 21)			CoNS (n = 06)		
	S rate (%)	R rate (%)	I rate (%)	S rate (%)	R rate (%)	I rate (%)	S rate (%)	R rate (%)	I rate (%)	S rate (%)	R rate (%)	I rate (%)	S rate (%)	R rate (%)	I rate (%)
P (10UI)	0.00	100.00	0.00	16.67	83.33	0.00	5.56	94.44	0.00	9.52	90.48	0.00	50.00	50.00	0.00
OX (1 μg)	93.94	6.06	0.00	83.33	16.67	0.00	61.11	38.89	0.00	33.33	66.67	0.00	66.67	16.67	16.67
FOX (30 μg)	93.94	6.06	0.00	83.33	16.67	0.00	61.11	38.89	0.00	47.62	52.38	0.00	83.33	16.67	0.00
AMC (20/10 μg)	100.00	0.00	0.00	94.44	5.56	0.00	94.44	5.56	0.00	80.95	19.05	0.00	83.33	16.67	0.00
GM (10 μg)	100.00	0.00	0.00	100.00	0.00	0.00	94.44	0.00	5.56	85.71	14.29	0.00	83.33	16.67	0.00
E (15 μg)	81.82	9.09	9.09	55.56	27.78	16.67	72.22	27.78	0.00	47.62	52.38	0.00	66.67	16.67	16.67
K (30 μg)	54.55	9.09	36.36	83.33	11.11	5.56	66.67	27.78	5.56	85.71	14.29	0.00	83.33	16.67	0.00
TE (30 μg)	54.55	45.45	0.00	44.44	55.56	0.00	55.56	44.44	0.00	47.62	52.38	0.00	50.00	50.00	0.00
VA (30 μg)	96.97	3.03	0.00	100	0.00	0.00	100.00	0.00	0.00	100.00	0.00	0.00	100.00	0.00	0.00
CL (2 μg)	90.91	6.06	3.03	61.11	33.33	5.56	94.44	0.00	5.56	95.24	0.00	4.76	100.00	0.00	0.00
RIF (5 μg)	96.97	3.03	0.00	88.89	11.11	0.00	88.89	11.11	0.00	85.71	9.52	4.76	100.00	0.00	0.00
STX (1.25/23.75 μg)	100.00	0.00	0.00	83.33	5.56	11.11	100.00	0.00	0.00	80.95	14.29	4.76	83.33	0.00	16.17

*P* penicillin, *OX* oxacillin, *FOX* cefoxitin, *AMC* amoxicillin + clavulanic acid, *GM* gentamicin, *E* erythromycin, *K* kanamycin, *TE* tetracycline, *VA* vancomycin, *CL* clindamycin, *RIF* rifampicin, *STX* trimethoprim/sulfamethoxazole, *S* susceptible, *R* resistant, *I* intermediate

Oxacillin and/or cefoxitin were confirmed as MRSA using E-test method (Liofilchem, Loc Piane Vomano, Italy).

#### Detection of resistance-associated genes by PCR

Genomic DNA was extracted using phenol/chloroform extraction method [32].

PCR amplifications were carried out for *aac*A-*aph*D*, erm*A*, erm*C*, tet*K and *tet*M, *bla*Z and *mec*A genes according to methods described previously [16, 33, 34], respectively.

The DNA fragments were visualized using an UV transilluminator (EC3, UVP BioImaging Systems, Cambridge, UK) and read using AlphaEaseFC software (Genetic Technologies Ltd., Fitzroy, Australia).

#### Statistical analysis

Possible relationships between the presence of resistance genes among *S. aureus* and CoNS isolated from food and clinical samples were statistically analysed using Fisher’s exact test. *P*-value of < 0.05 was considered as statistically significant difference.

### Results

Ninety-six staphylococci were isolated, identified and classified as 51 staphylococci (33 *S. aureus* and 18 CoNS) from food samples, 27 staphylococci (21 *S. aureus* and 6 CoNS) from community-acquired clinical samples and 18 *S. aureus* from hospital-acquired clinical samples, respectively.

Out of 51 staphylococci isolated from food samples, 49 staphylococci (33 *S. aureus* and 15 CoNS) were resistant to at least one tested antibiotic (96.1%) (Table 1). The resistance rates to penicillin and tetracycline were 94.1% and 49.0%, respectively. Two *S. aureus* (6.1%) and three CoNS (16.7%) were phenotypically resistant to methicillin by disc diffusion test while MIC determination by E-test confirmed only one isolate as MRSA and another as methicillin-resistant CoNS.

Vancomycin resistance was detected in one *S. aureus* (3.0%). The resistance rates of CoNS to clindamycin and erythromycin were 33.3% and 27.8%, respectively. All staphylococci isolated from food samples were susceptible to gentamicin.

The antimicrobial resistance rates were remarkably higher in clinical isolates than in staphylococci isolated from food samples (Table 1). The resistance rates of *S. aureus* and CoNS isolated from clinical samples to penicillin were 92.3% and 50.0%, respectively while those to tetracycline were 48.7% and 50.0% for *S. aureus* and CoNS, respectively. The prevalence of MRSA obtained by disc diffusion test was 38.9% and 52.4% for hospital and community-acquired isolates, respectively. However, the detected MRSA rate by E-test was 11.1% and 23.8%. *Staphylococcus aureus* showed considerable resistance to erythromycin for both hospital-acquired (27.8%) and community-acquired isolates (52.4%). *Staphylococcus aureus* isolates from hospital were susceptible to gentamicin, however, those isolated from community infection showed 14.3% resistance to gentamicin. All clinical staphylococci isolates were susceptible to vancomycin.

The antibiotic resistance-associated genes were demonstrated in Table 2. In food isolates *tet*M was the most prevalent gene detected in *S. aureus* and CoNS with 66.7% and 88.9%, respectively. Both *tet*M and *tet*K genes were detected in 11 *S. aureus* (33.3%) and 4 CoNS (22.2%). In food samples, *mec*A gene was observed in 15 *S. aureus* (46.9%) and 10 CoNS (88.9%) isolates, respectively. Six *S. aureus* (18.2%) and 3 CoNS (16.7%) isolates harboured *aac*A-*aph*D gene associated with gentamicin-resistance. Fifteen *S. aureus* (46.9%) and 10 CoNS (88.9%) isolates possessed *bla*Z gene encoding resistance to penicillin. No significant difference for the detection of antibiotics resistance genes was observed between *S. aureus* and CoNS isolated from food samples (P > 0.05).

**Table 2:** Distribution of antimicrobial resistance genes of food and clinical isolates

Antibiotic agent	Target genes	Food isolates (n = 51)		*P* value	Clinical isolates (n = 45)			*P* value
		*S. aureus* (n = 33)	CoNS (n = 18)		Hospital acquired isolates (n = 18)	Community acquired isolates (n = 27)		
					*S. aureus* (n = 18)	*S. aureus* (n = 21)	CoNS (n = 06)	
Tetracycline	*tet*M	22 (66.67%)	16 (88.89%)	0.103	10 (55.56%)	17 (80.95%)	2 (33.33%)	0.162
	*tet*K	13 (39.39%)	4 (22.22%)	0.351	6 (33.33%)	11 (52.38%)	1 (16.67%)	0.333
Erythromycin	*erm*A	0 (0.00%)	0 (0.00%)	ND	0 (0.00%)	0 (0.00%)	0 (0.00%)	ND
	*erm*C	2 (6.06%)	1 (5.56%)	1.000	1 (5.56%)	5 (23.81%)	0 (0.00%)	0.189
Gentamicin	*aac*A-*aph*D	6 (18.18%)	3 (16.67%)	1.000	3 (16.67%)	9 (42.86%)	2 (33.33%)	0.095
Penicillin	*bla*Z	15 (46.87%)	10 (88.89%)	0.565	6 (33.33%)	15 (80.95%)	2 (33.33%)	0.025
Methicillin	*mec*A	15 (46.87%)	10 (88.89%)	0.565	17 (94.44%)	19 (90.48%)	6 (100%)	1.000

Regarding staphylococci from clinical samples*, mec*A was the most detectable resistance-associated gene in *S. aureus* originated from the hospital with 17 isolates (94.4%) and in the community with 19 isolates (90.5%). The *tet*M gene was found in 10 hospital isolates (55.6%) and 17 community-acquired isolates (81.0%). The *tet*K and *tek*M genes were detected together in 10 *S. aureus* isolates (25.6%). Six *S. aureus* from the hospital (16.7%) harboured *erm*C gene. The *aac*A-*aph*D gene was found by PCR in 12 *S. aureus* isolates (3 from hospital and 9 were community-acquired). The *bla*Z gene detection rate was significantly higher in hospital-acquired *S. aureus* with 15 isolates (81.0%) than in community-acquired isolates with only 6 (55.6%) (P = 0.025). No statistical differences were observed for the detection of remaining antibiotic genes.

The correlation between phenotypic resistance and detection of resistance-associated genes for food and clinical isolates was demonstrated in Table 3. In *S. aureus* from food samples correspondence between phenotypic resistance and associated genes was 45.5% and 50.0% concerning penicillin and methicillin, respectively. For tetracycline resistant isolates 46.7% and 53.3% harboured *tet*K and *tet*M, respectively. Both genes were detected in 33.3%. In clinical isolates the correlation between resistance and *mec*A gene was very high with 100% and 92.8% in hospital-acquired and community-acquired *S. aureus*.

**Table 3:** Correlation between phenotypic resistance and detection of resistance-associated genes of food and clinical isolates

Antibiotic	Gene(s)	Food isolates (n = 51)				Clinical isolates (*S. aureus* n = 39)			
		*S. aureus* (n = 33)		CoNS (n = 18)		Hospital-acquired isolates (n = 18)		Community-acquired isolates (n = 21)	
		Phenotypic resistance	Gene detection	Phenotypic resistance	Gene detection	Phenotypic resistance	Gene detection	Phenotypic resistance	Gene detection
Penicillin	*bla*Z	33	15 (45.5%)	15	7 (46.7%)	17	6 (35.3%)	19	13 (68.4%)
Methicillin	*mec*A	2	1 (50.0%)	3	1 (33.3%)	7	7 (100%)	14	13 (92.8%)
Gentamicin	*aac*A-*aph*D	0	0	0	0	0	0	3	1 (33.3%)
Erythromycin	*erm*C	3	0	1	1 (100%)	5	1 (20.0%)	11	4 (36.4%)
Tetracycline	*tet*M	15	8 (53.3%)	10	9 (90.0%)	8	4 (50.0%)	10	9 (90.0%)
	*tet*K		7 (46.7%)		2 (20.0%)		4 (50.0%)		8 (80.0%)
	*tet*M + *te*tK		5 (33.3%)		2 (20.0%)		2 (25.0%)		7 (70.0%)

### Discussion

The results of this study showed less prevalence of *S. aureus* resistant to oxacillin in food isolates (6.1%) compared to those reported previously 67.5% [35], 38.0% [36] and 17.3% [37].

The prevalence of oxacillin-resistance in clinical isolates was slightly similar to previous study in Algiers [38] but lower than another study in eastern Algeria [39].

*Staphylococcus aureus* from food and clinical samples were phenotypically oxacillin-resistant with 6.1% and 46.2%, respectively. From isolates 46.8% and 92.3% of them harboured the *mec*A gene, respectively. In contrast, 38.0% of Portuguese *S. aureus* strains from food samples were resistant to oxacillin but only 0.7% of them harboured *mec*A gene [36] and in Libya, *mec*A gene was not detected by PCR in MRSA isolated from hospitalized patients [40].

In Morocco, Kenya, Nigeria and Cameroun relatively high occurrence rates of MRSA were reported [41]. In Algeria, the majority of MRSA did not represent a high multidrug resistance rate [41, 42].

All isolates from food were resistant to penicillin which was in agreement with previous reports [37, 43, 44] but significantly higher than reported in other studies [35, 36, 45].

*Staphylococcus aureus* isolated from clinical samples were highly resistant to penicillin (> 90.0%) which is in agreement with previous studies [46–49].

The presence of the *bla*Z gene did not influence production of β-lactamase alone [50, 51]. Here, not all penicillin-resistant *S. aureus* isolates from food (45.5%) and clinical samples (52.8%) possessed *bla*Z gene.

All *S. aureus* isolates from food samples were susceptible to gentamicin, which agrees with several reports [36, 52, 53]. In all clinical isolates, the resistance rate of *S. aureus* to gentamicin was 7.7% which was similar (7.0%) with a study performed in Algeria [25]. In Algeria, no resistance to gentamicin was found in staphylococci in milk [54, 55]. One *S. aureus* isolate exhibiting phenotypic resistance to gentamicin harboured the *aac*A-*aph*D gene which is in contrast to previous studies [16, 33]. In this study, *aac*A-*aph*D was detected more often in staphylococci isolates from clinical (31.1%) than those of food origin (17.6%).

*Staphylococcus aureus* isolates from food were slightly resistant to erythromycin (9.1%) which was higher than 5% reported previously in Portugal [36], but significantly lower than detected in China and Turkey [37, 56].

The resistance of clinical *S. aureus* to erythromycin was higher than reported previously in Algerian hospitals [25]. The *erm*A gene encoded erythromycin-resistance could not be detected in both *S. aureus* and CoNS from clinical and food origin. In contrast, high rate of *erm*A and *erm*C genes in MRSA isolates from hospitalized patients was determined [57]. The correlation between phenotypic susceptibility testing and detection of *erm*C gene was 31.3% which was lower than obtained previously [16, 33]. In Libya, *erm*A and *erm*C genes could not be detected in erythromycin-resistant *S. aureus* [40].

The resistance of *S. aureus* isolated from food samples to tetracycline was higher than reported formerly in China [56], in Italy [6], in Portugal [36] and in Turkey [45]. Tetracycline resistance of *S. aureus* isolated from clinical samples was considered lower than reported before [25, 48]. There was no significant difference between tetracycline resistance in staphylococci isolated from food and clinical samples. In contrast, a higher resistance rate to tetracycline in food isolates compared to clinical isolates was described [52]. The *tet*K and *tet*M genes were detected in 53.3% and 60.0% in resistant *S. aureus* from food samples, respectively. In clinical *S. aureus* isolates, the correlation rate between phenotypic resistance and prevalence of *tet*M and *tet*K genes was 72.2% and 66.7%, respectively. The discrepancy between phenotypic resistance to tetracycline and resistance determinants may attributed to other mechanisms [15, 58].

Vancomycin was the most effective antimicrobial agent against MRSA [41, 59]. In this study, three *S. aureus* strains from food showed resistance to vancomycin. In Turkey, 21.7% of *S. aureus* isolated from food samples were resistant to vancomycin [37].

Most of CoNS isolates were resistant to penicillin, tetracycline and erythromycin. In addition, 16.7% of CoNS isolated from food samples were confirmed phenotypically as methicillin-resistant CoNS (MR-CoNS). The *mec*A gene was detected in 75.0% of isolated CoNS which was in agreement with previous study [33]. The *erm*C gene was detected only in one CoNS isolate (5.6%) which was in accordance with formerly reports [33, 60]. In contrast, staphylococci isolated in Turkey showed high detection rate of *erm*C and *erm*A [61, 62].

In conclusion, the high prevalence of resistance to penicillin, tetracycline and erythromycin was particularly alarming. The knowledge about antibiotic resistance of staphylococci originated from daily food from markets and of community-acquired *S. aureus* is not fully addressed in Algeria, yet. Information concerning resistance in CoNS is very limited in this country. Vancomycin-resistant isolates were found in food isolates not in clinical ones. This study considered a first impression on existing situation concerning antibiotic resistance on staphylococci from food and clinical sources. Surveillance on antibiotic resistance and characterization of staphylococci in Algeria is mandatory.

## Limitations

This study used exclusively samples from cities of Médéa and Ain Defla provinces, Algeria which limits the generalisation of the results.

## Abbreviations

CoNS: coagulase negative Staphylococcus
PCR: polymerase chain reaction
P: penicillin
OX: oxacillin
FOX: cefoxitin
AMC: amoxicillin + clavulanic acid
GM: gentamicin
E: erythromycin
K: kanamycin
TE: tetracycline
VA: vancomycin
CL: clindamycin
RIF: rifampicin
STX: trimethoprim/sulfamethoxazole
S: susceptible
R: resistant
I: intermediate
